# Text mining for contexts and relationships in cancer genomics literature

**DOI:** 10.1093/bioinformatics/btae021

**Published:** 2024-01-22

**Authors:** Charlotte Collins, Simon Baker, Jason Brown, Huiyuan Zheng, Adelyne Chan, Ulla Stenius, Masashi Narita, Anna Korhonen

**Affiliations:** Language Technology Laboratory, Theoretical and Applied Linguistics, Faculty of Modern and Medieval Languages and Linguistics, University of Cambridge, Cambridge CB3 9DA, United Kingdom; Language Technology Laboratory, Theoretical and Applied Linguistics, Faculty of Modern and Medieval Languages and Linguistics, University of Cambridge, Cambridge CB3 9DA, United Kingdom; Language Technology Laboratory, Theoretical and Applied Linguistics, Faculty of Modern and Medieval Languages and Linguistics, University of Cambridge, Cambridge CB3 9DA, United Kingdom; Institute of Environmental Medicine, Karolinska Institutet, 171 77 Stockholm, Sweden; Cancer Research UK Cambridge Institute, Li Ka Shing Centre, University of Cambridge, Cambridge CB2 0RE, United Kingdom; Institute of Environmental Medicine, Karolinska Institutet, 171 77 Stockholm, Sweden; Cancer Research UK Cambridge Institute, Li Ka Shing Centre, University of Cambridge, Cambridge CB2 0RE, United Kingdom; Language Technology Laboratory, Theoretical and Applied Linguistics, Faculty of Modern and Medieval Languages and Linguistics, University of Cambridge, Cambridge CB3 9DA, United Kingdom

## Abstract

**Motivation:**

Scientific advances build on the findings of existing research. The 2001 publication of the human genome has led to the production of huge volumes of literature exploring the context-specific functions and interactions of genes. Technology is needed to perform large-scale text mining of research papers to extract the reported actions of genes in specific experimental contexts and cell states, such as cancer, thereby facilitating the design of new therapeutic strategies.

**Results:**

We present a new corpus and Text Mining methodology that can accurately identify and extract the most important details of cancer genomics experiments from biomedical texts. We build a Named Entity Recognition model that accurately extracts relevant experiment details from PubMed abstract text, and a second model that identifies the relationships between them. This system outperforms earlier models and enables the analysis of gene function in diverse and dynamically evolving experimental contexts.

**Availability and implementation:**

Code and data are available here: https://github.com/cambridgeltl/functional-genomics-ie.

## 1 Introduction

Scientific advances depend on collective learning, or the ability to communicate, record, and progressively build on knowledge over time. Since the first publication of the human genome in 2001 (Lander *et al.* 2001, Venter *et al.* 2001), the rapid development of new technologies in the biomedical field of genomics has led to the publication of vast numbers of studies seeking to decode the complex relationship between genotype (genetic constitution) and phenotype (the physical and behavioural attributes resulting from a genotype) (Przybyla and Gilbert 2022). The function of individual genes varies with context such that the same gene may perform variably in different species, tissues, disease states, and experimental approaches. Genomics has the potential to revolutionize our understanding and treatment of human diseases, however, the quantity of literature available is too large for researchers to read and analyse manually. Most biomedical research is stored and accessed through the online biomedical database, PubMed, which already contains more than 34 million citations and is continuing to grow exponentially.

Text mining (TM) can facilitate information extraction from large volumes of literature by turning unstructured, human-generated language into structured data (Renganathan 2017). Biomedical TM requires both the means of recognizing biological entities and strategies for identifying contextual details that specify their roles in experiments. TM employs Natural Language Processing (NLP) technology, which trains neural networks of machine-learning algorithms on specialized texts to develop domain-specific language representation models. Recent developments in NLP, such as Transformers (Lee *et al.* 2020), are used for general biomedical TM tasks. Here, we seek to develop the first TM methodology that is specifically customized for extracting the contexts of genomics experiments. We develop a Named Entity Recognition (NER) model as well as a relation extraction model to allow the accurate identification and extraction of methods of gene perturbation, phenotypes, and important details of experiment context such as species, cell type, and experiment setting (*in vitro* or *in vivo*). Previous work has focused on extracting genotypes and associated phenotypes (Xing *et al.* 2018, Sousa *et al.* 2019) from scientific literature without linking them to associated contexts, thereby missing key information.

We focus on the functional genomics of cancer, a domain in which TM could have particularly high impact. Cancer is one of the leading causes of death worldwide, with 19.3 million new cases and 10 million deaths estimated to occur in 2020 alone (Sung *et al.* 2021). It is therefore the subject of intensive research efforts; as of February 2023, a search of the PubMed biomedical database for the term ‘cancer’ returned some 4 795 969 citations. Cancer genomics explores how DNA sequences and gene expression patterns differ between healthy cells and cancer cells. Genetic differences define the distinct types of cancers and cancer cell subpopulations and also dictate their different susceptibilities to pharmacogenetic intervention (Berger and Mardis 2018). A key focus of cancer genomics research is the identification of genes which regulate cell death in different cell types and contexts. By pinpointing those genes that regulate cell death in cancer cells, but not in healthy cells, it may be possible to develop new drugs that can precisely target cancer cells whilst sparing healthy cells from harmful toxic effects. Technology that can automatically extract and collate contextualized genomics data from the entire PubMed corpus could help scientists identify the most promising new gene targets and thereby accelerate the development of cancer therapeutics.

## 2 Background

### 2.1 Cancer genomics and cell death

Understanding the genetics of cell death is a major focus of the cancer genomics field. The balance between the rate of cell division and the rate of cell death forms the essential mechanism by which multicellular organisms maintain the correct number of cells in their bodies. Failure to properly regulate this process can lead to uncontrolled cell proliferation and cancer.

Cell death can occur either as a result of external assault or through a process of ‘cell suicide’ in which genetically regulated internal programmes drive the active pursuit of cell death in order to clear damaged or unwanted cells from the body [reviewed in Strasser and Vaux (2020)]. Cell death results from the failure of essential metabolic functions [reviewed in Galluzzi *et al.* (2018)] and can occur through different mechanisms distinguishable by distinct morphological and genetic characteristics. Some commonly described cell death types are ‘Apoptosis’ (Kerr *et al.* 1972), a regulated form of cell death that is characterized by cytoplasmic shrinkage, fragmentation of nuclei, chromatin condensation, and membrane blebbing, ‘Autophagy’ or ‘self-eating’ (Ohsumi 2014), which is a an alternative form of cell death in some contexts and is characterized by the development of double-membraned autophagosomes (Ohsumi 2014), and ‘Necrosis’, which can result from either external trauma or internal activation and lacks the distinctive morphological features of apoptosis and autophagy [reviewed in Galluzzi *et al.* (2018)]. Other cell death subtypes include distinct forms specific to different cell types and contexts. Of particular significance for cancer research, the different mechanisms of cell death are regulated by different groups of genes, described in Supplementary Table S1.

The different forms of cell death have multiple roles in the pathogenesis and treatment of cancers. Cancer cells are characterized by their abilities to evade the normal mechanisms of cell death and to proliferate in an uncontrolled way, resulting in tumours. In addition, death of pre-cancerous cells within a tumour can lead to the release of mitogenic factors, which induce compensatory proliferation in adjacent cells (Labi and Erlacher 2015, Celis *et al.* 2022). Cancer treatments include the use of radiation or drugs to induce death of cancer cells, whilst aiming to minimize harmful side-effects. The ability of subsets of tumour cells to resist cell death, however, can lead to the re-population of tumours with more aggressive clones and subsequent cancer progression. In addition, a subset of surviving tumour cells may adopt a senescence phenotype and subsequently promote wide-ranging adverse effects including inflammation, cardiac dysfunction, and cancer recurrence (Demaria *et al.* 2017). It is therefore of great interest to develop drugs that can target specific genes to kill therapeutically significant subsets of cancer cells without toxicity to normal, healthy cells. An expansive body of research has aimed to generate a detailed understanding of the types of cell death that occur in cancer and non-cancer cell types and of the different groups of genes that regulate them. To maximize the value of this literature to the cancer research and genomics communities, we need to develop tailored TM methodology that can carry out large-scale extraction of the actions of genes in different contexts and cell death subtypes. The data generated could identify novel genes that regulate cell death specifically in cancer cells, thereby allowing the development of targeted cancer drugs and opening up new therapeutic avenues.

### 2.2 Biomedical TM

TM is a specialized field in the biomedical domain and has been used to address many different challenges in biology, cancer research, and drug discovery. Several methods and web-based resources for the analysis of genomics data have been developed, including LitMiner (Demaine *et al.* 2006), GeneMANIA (Warde-Farley *et al.* 2010), OnTheFly (Pafilis *et al.* 2013), SIGNOR (Perfetto *et al.* 2016), Enrchr (Kuleshov *et al.* 2016), Cancer Hallmarks Analytics Tool (Baker *et al.* 2016, 2017), LION-LBD (Pyysalo *et al.* 2019), GENETEX (Miller and Shalhout 2021), Cancer Dependency Map (Shimada *et al.* 2021), and the Database of Essential Genes (DEG 15) (Luo *et al.* 2021). While useful for many purposes, none of these existing resources are specifically adapted for extraction of functional genomics data from journal papers. They are limited in their ability to extract context from semantically rich texts, searches can be performed for gene(s) but not for phenotypes or details of experiment context, and their results are infrequently updated and therefore quickly become out-of-date in the fast moving genomics field.

Biomedical TM aims to both recognize biological entities and to capture the ways in which two or more entities interact, in relationships commonly termed ‘bio-events’(Ananiadou *et al.* 2015). To extract the most important details of genomics experiments, it is necessary to identify gene names and symbols, the methods by which genes are experimentally perturbed, the phenotypes that result from gene perturbations and the contexts in which experiments are carried out. Resources are available for the automatic recognition and grounding of genes, such as the Gene Ontology database (Ashburner *et al.* 2000), and of phenotypes, such as the Human Phenotype Ontology (HPO) (Groza *et al.* 2015), but there are no equivalent resources to aid recognition of important contextual details, such as methods of gene perturbation, species, cell or tissue type, and experimental approach. Of note, the names of cell lines and gene constructs are often highly individualized and may appear in only a small number, or in some cases a single paper(s). Further, the co-occurrence of two or more entities in the same sentence is not always evidence of their interaction (Chun *et al.* 2006), and some entities may interact with multiple other entities which could be either adjacent or distant.

We aim here to address the significant challenges of accurately identifying relevant cancer genomics entities in large volumes of literature and of extracting these entities together with their complex inter-relationships. To this end, we develop a new corpus of biomedical literature that has been labelled with both genes and phenotypes and important details of experiment context. We apply the latest machine-learning algorithms, such as Transformers (Devlin *et al.* 2018) and BioBERT (Lee *et al.* 2020), to construct two transformer models that are specifically targeted towards identifying cancer functional genomics entities and linking these entities to relevant context identified in text. The novel ability to extract context represents a significant advance in biomedical TM methodology and extends researchers’ ability to obtain the most highly relevant genomics information from literature.

## 3 Materials and methods

### 3.1 Corpus annotation

#### 3.1.1 PubMed literature retrieval

To obtain relevant texts, we retrieved abstracts from eight PubMed-listed journals that are prominent within the cancer or cell death fields; these were ‘Apoptosis’, ‘Autophagy’, ‘Cancer Cell’, ‘Cancer Research’, ‘Cell’, ‘Cell Death and Differentiation’, ‘Cell Death and Disease’, and ‘Genes and Development’. Using the PubMed Advanced Search tool, we selected abstracts that included one of these titles as a journal name but excluded ‘review’ as a publication type. The first 100 abstracts (listed by PubMed on 12 May 2021) for each journal were downloaded. Abstracts were manually processed into a total of 800 individual text files, in which only the article title and the main body of the abstract were included, with the author list and all other extraneous text being removed.

#### 3.1.2 Cell death, cancer, and genetics terminology

To systematically extract the key elements of experiment descriptions from texts, we focused on words and phrases defined by four main categories. These were chosen through discussion with experts in the cancer and cell death research field and capture the most relevant details of cancer and genomics experiments. The four categories were: ‘Perturbing actions’, ‘Contexts’, ‘Phenotypes’, and ‘Effects’. (i) Perturbing actions are defined experimental manipulations that relate to the regulation of any named gene or genes. We included gene names, symbols, and recognized aliases, using www.genecards.org as a reference. (ii) Contexts are the experimental models subjected to a Perturbing action, e.g. human patients, laboratory animals or cultured primary cells and cell lines. (iii) Phenotypes are the induced changes in the physical characteristics and/or behaviour of the cell or organism that result from a Perturbing action. We restricted this category to terms specific to cell death, terms specific to cancer development and to some common cell biology terms, which can relate to the behaviour of cells in both cancerous and healthy tissues. The list of terms was chosen with reference to relevant literature (Galluzzi *et al.* 2018) and after discussion with experts from the cell death and cancer field at Cancer Research UK Cambridge Research Institute, UK. (iv) Effects are the direction in which a Phenotype is regulated, i.e. positively or negatively. A full list of markable words and phrases is shown in Table 1.

**Table 1.** Definitions of each entity category and corresponding assertions for each category.

Entity type	Definition	Assertions used to label marked entities
Perturbing action	Defined experimental manipulations that relate to the regulation of a named gene or genes.	Gene loss-of-function, gene gain-of-function, RNAi/knockdown, pharmacological inhibition, pharmacological augmentation, other.
Context	The cells or organisms that are the subject of an experimental manipulation.	Patient, organism, tissue/organ, neoplasm, graft, xenograft, cells, transformed cells, organoid, *in vitro*, *in vivo*.
Phenotype	The induced changes in the physical characteristics and/or behaviour of the organism or cell.	Cell death terms: apoptosis, anoikis, autophagy, cell death, entosis, ferroptosis, mitophagy, necroptosis, necrosis, oncosis, and pyroptosis. Cancer terms: metastasis, transformation, tumour-growth, tumourigenesis, tumour initiation, tumour progression, and tumour regression. General cell biology terms: adhesion, cell cycle arrest, cell growth, cell survival, colony formation, differentiation, epithelial-mesenchymal transition, invasion, migration, proliferation, quiescence, self-renewal, and senescence.
Effect	If a phenotype is regulated positively or negatively.	Positive, negative, regulates, rescues, no effect.

#### 3.1.3 Annotation of texts

The annotation was carried out by a biologist with 9 years of postgraduate experience in cell biology experimental research. The Multi-Annotation Environment (MAE) annotation tool was used (Stubbs 2011, Rim 2016) (http://keighrim.github.io/mae-annotation/). The framework of the annotation task was defined using a custom DTD file.

Abstracts were annotated in list order following retrieval from PubMed. Only abstracts linked to primary research articles were annotated. Reviews, commentaries, and editorial articles were excluded from the task. Annotation was carried out at whole text level; however, only the parts of abstracts containing descriptions of experiments were marked. We did not include text that formed part of the introduction or discussion, nor did we mark the text of purely descriptive studies or parts of studies, such as accounts of gene expression patterns.

Annotation was carried out at the level of whole abstracts. Words or continuous phrases were marked when they both (i) formed part of the description of an experiment and (ii) corresponded to one of the terms of interest listed in Table 2. Descriptions of experiments (though not individual entities) sometimes spanned multiple sentences. All marked Perturbing actions included a gene name or symbol, and marked Contexts included names of cell types, cell lines, species, and breeding lines when present. In some abstracts, words or phrases from all four categories were marked; in other abstracts it was only possible to mark words or phrases from some of the categories. The total number of marked entities in the corpus was 10 458.

**Table 2.** An example of marking and labelling the description of an experiment.^a^

Marked text segment	Category	Assertion
Silencing Lnc-EPIC1 by siRNA	Perturbing action	RNAi/knockdown
Inhibit	Effect	Negative
Cell growth	Phenotype	Cell growth
Colony formation	Phenotype	Colony formation
PC cells	Context	Cells
Induced	Effect	Positive
G1/S cell cycle arrest	Phenotype	Cell cycle arrest
Apoptosis	Phenotype	Apoptosis
PC cells	Context	Cells

a Silencing Lnc-EPIC1 by siRNA targeting could significantly inhibit the cell growth and colony formation ability of PC cells and induced G1/S cell cycle arrest and apoptosis in PC cells.

The MAE tool allows the addition of labels, or ‘assertions’, to marked entities. Lists of assertions relating to each category of markable entity (Perturbing actions, Contexts, Phenotypes, and Effects) were defined within the DTD document and appeared as drop-down lists during the annotation process. Each marked entity was labelled with the appropriate assertion to identify its meaning. For example, ‘cell’, ‘myoblast’, and ‘keratinocyte’ were each labelled with the assertion ‘cell’, as all these terms describe cell types; ‘Rag2 -/-’ was labelled with the assertion ‘gene loss-of-function’, as ‘-/-’ denotes knockout of the Rag2 gene in both alleles. An example of marking and labelling the text of an experiment description is shown in Table 2.

#### 3.1.4 Linking marked entities

**Figure 1.**
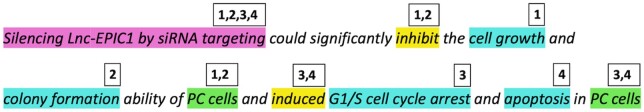
An example of marking and linking entities. Marked entities (shaded) are assigned to one of four categories: Perturbing actions i.e. Silencing Lnc-EPIC1 by siRNA targeting (purple), Contexts i.e. PC cells (green), Effects i.e. inhibit; induce (yellow) or Phenotypes i.e. cell growth; colony formation; G1/S cell cycle arrest; apoptosis (blue). There are four groups (1, 2, 3, and 4) of four linked (related) entities. Each linked group contains one entity from each category, labelled with the same number. Some entities participate in more than one group and are therefore labelled with more than one number.

Many abstracts contained multiple examples of Perturbing actions, Contexts, Phenotypes, and Effects. We created Link tags to record links between four marked entities (one from each category) that formed part of the description of the same experiment. Because the descriptions of experiments sometimes spanned more than one sentence, the four entities in each linked group were sometimes derived from more than one sentence. Where a description of one experiment generated multiple marked entities within one or more categories, we created multiple Link tags. An example of creating Link tags between marked entities is shown in Fig. 1. The total number of entities that were both marked and linked was 4697.

#### 3.1.5 Statistics of annotated data

To measure the precision of the annotation process, a secondary annotator with cell biology experience undertook annotation of a subset of 30 abstracts (numbers 561–590). The results of the primary annotator and the secondary annotator were compared using a statistical tool within the MAE program to calculate values for Krippendorf’s Alpha-U, where a value of one indicates complete agreement. Good text marking (the correct identification of markable entities) agreement was achieved with a mean of 0.79 across all four categories (with individual values for Perturbing actions of 0.72, for Contexts of 0.82, for Phenotypes of 0.86, and for Effects of 0.79). Disagreement between annotators most often arose from the omission of whole entities rather than variation in entity boundaries, though examples of both occurred. We also achieved generally good agreement for labelling (attaching correct assertions) of marked entities within all four categories, with Krippendorf’s Alpha-U values for Perturbing actions of 0.71, for Contexts of 0.74, for Phenotypes of 0.84, and for Effects of 0.68. For both text marking and entity labelling, the greatest precision was found within the Phenotypes category.

### 3.2 Natural Language Processing

#### 3.2.1 Data pre-processing

The MAE tool XML output was converted to JSON via the python standard library. This results in the text of each abstract accompanied by lists of tags for each category. Each tag has a unique id and contains the span of text and label. There are also lists of linked entities, each with a unique id, the tag ids, and text spans for the tags. The per-tag data were converted to per-word data for each word in each abstract. Each datum contained the word, the tokenization of that word, and any tags it had. Each tag entry contained the tag id, all link ids relating to that tag, the schema label, and the tag label itself. We used the **B**eginning **I**nside, **O**utside (BIO) format for tagging—words at the start of a tagged span had the ‘B’ schema label for that tag with ‘I’ for subsequent tags, and an ‘O’ to denote tokens that were not tagged.

For each word, its tags were copied onto each token, and then each token was converted to an id by the tokenizer before being input into the model. Label outputs were represented as one-hot encoded vectors, with one vector for each category of label and an additional one whose elements were the tag categories themselves. For each label the ‘B’ and ‘I’ versions had separate elements in the one-hot representation. Links were represented as an upper triangular matrix with element *ij* (where i≤j) being one if the *i*th and *j*th tokens of the abstract were linked in any tag, and zero if not.

#### 3.2.2 Machine learning

**Figure 2.**
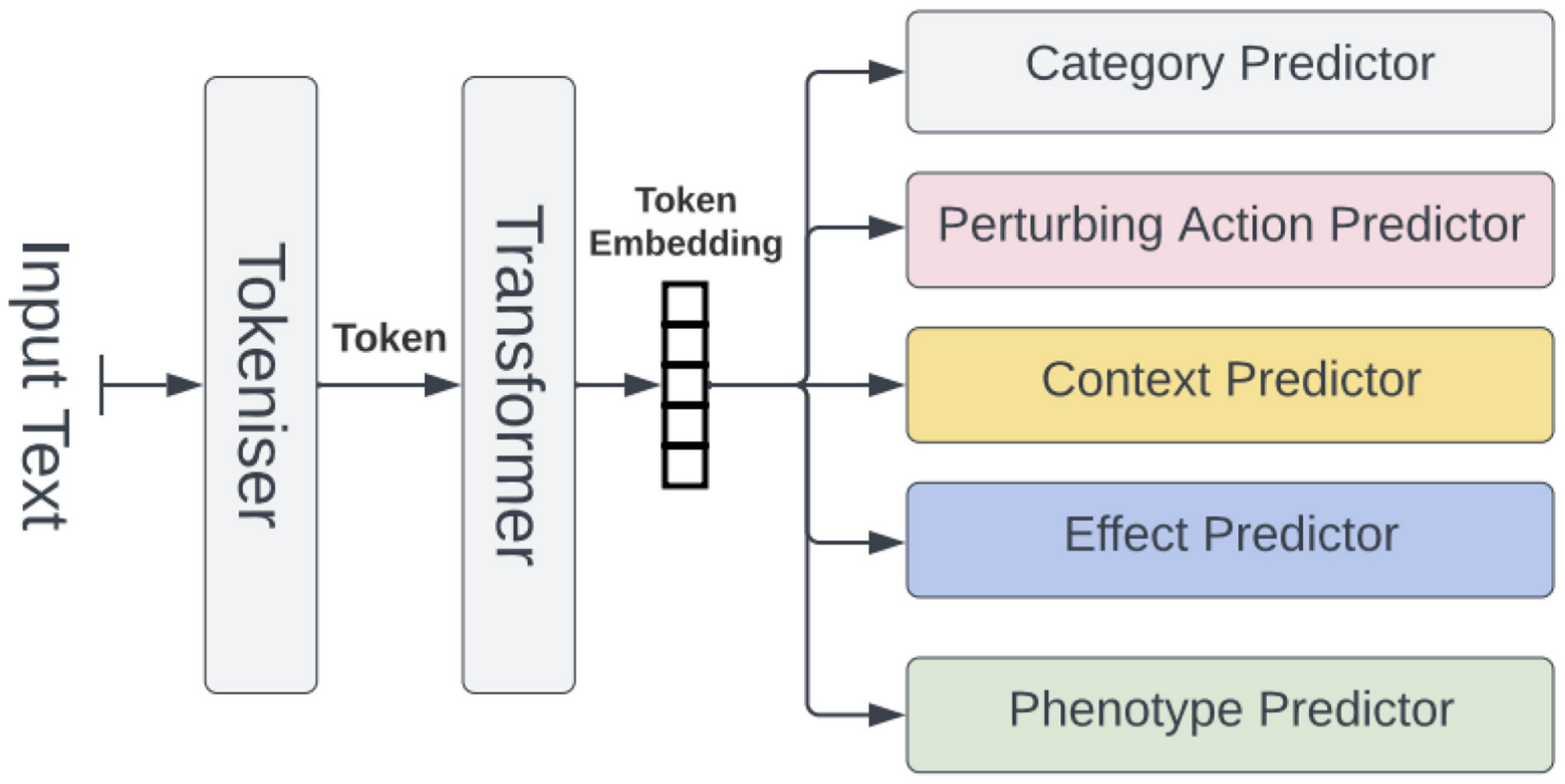
Architecture of the tag prediction neural model. A transformer produces an embedding of each token, which is fed into several different predictors. Each predictor is a linear classifier. The Category Predictor determines which of the four categories (Perturbing action, Context, Effect, and Phenotype) a token is labelled, while the four specific category predictors predict an assertion term under the given category, see Tables 1 and 2.

**Figure 3.**
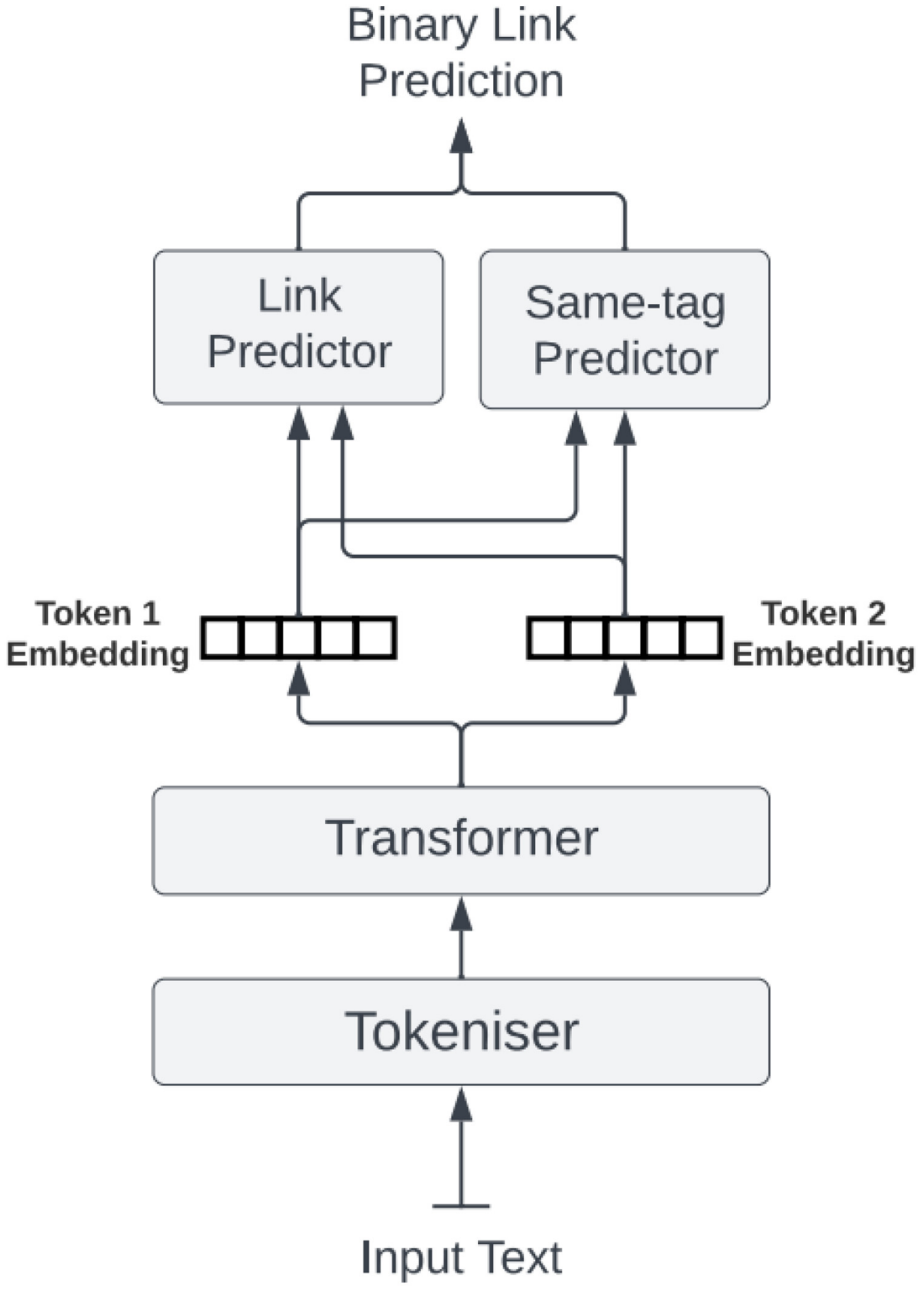
The link prediction model makes a binary prediction whether two entities that appear in the text should be associated with each other. The model is composed of two predictors (linear classifiers): the Link Predictor determines if two tokens are part of tags, which are linked, whilst the same-tag predictor determines if two tokens are a part of the same entity tag. Each predictor takes a concatenated pair of embeddings from the transformer as input.

We used two neural network models. The first (Fig. 2) is a NER model that tags spans of text with one of the four category labels (Perturbing Action, Context, Effect, and Phenotype), as well as assigns a relevant assertion listed in Table 1. The second model (Fig. 3) links the relevant entities identified by the NER model. For example: a perturbing action can be linked to an effect, a phenotype etc., as illustrated in the example shown in Fig. 1.

Both models consist of two parts. The first is a fine-tuned pre-trained BioBERT transformer (Lee *et al.* 2020) and the second is a set of predictors. Each predictor is a linear classifier—a dense layer followed by a softmax non-linearity. The category predictor decides which of the four main labels to assign to the token (or predict a non-assignment); likewise; for each of the categories there is a specific head to predict an assertion for that category. The heads were applied to each of the token embeddings produced by the transformer to predict the label for that token. We used cross-entropy loss function for training.

It was found that the inclusion of a category predictor improved training by increasing the effective amount of training data for the transformer.

The linking model has two predictors, one for whether two tokens are part of tags, which are linked, and one for whether or not they are part of the same tag. Since we are using a BIO schema for our tags, this latter predictor is not required since this information is encoded into the schema. Thus, results on its performance will not be reported. However, it was found that the inclusion of this second predictor aided the fine-tuning of the transformer, thus improving the accuracy of the first predictor, by increasing the effective amount of training data for the transformer fine-tuning. During the training process we concatenated every possible pair of token embedding vectors. The model predicts if tokens were linked via binary classification. We trained the model using a binary cross-entropy objective.

For both models, we allowed the transformer model’s parameters to be updated during the training process. As the transformers operate on the token-level they produce token-level classifications. The MAE tool operates on the word level, so outputs on tokens were converted to outputs on words by applying the prediction from the first token of the word. Other methods, such as averaging the predictions or taking a maximum prediction across all tokens, gave similar results so the simpler method was favoured.

We experimented with different baseline pre-trained transformer models and found that BioBERT to be the strongest for both the tagging and linking tasks. We also considered having each predictor with its own transformer base but early experiments showed this to be less effective as there has little training data for each specific category. We tried this again with our final architecture and hyperparameters and found it to perform similarly. Having multiple predictors on one transformer also reduces the number of transformers needing to be fine-tuned, thus training far quicker.

In order to mitigate the effects of having a small number of positive examples in the data, loss balancing was employed in both models, where losses for infrequent tags were given a higher weighting; this includes the ‘None’ tag label.

## 4 Results

### 4.1 Intrinsic evaluation

The data were split randomly to get a 10% size holdout final testing set. The remaining data were used in *k*-fold (*k* = 4) cross-validation, with hyperparameters being optimized for validation score. Due to the amount of data, the testing set was small so both testing and cross fold validation accuracy will be reported to give a clear indication of performance.

The linking model was trained, validated, and tested using the ground truth tags to avoid tag errors affecting the evaluation of the linking model’s accuracy. We measured the performance using standard metrics: precision, recall, and $F_{1}$ score. We averaged the scores across our cross-validation folds. We determined a true positive (TP) prediction when the tagging model correctly predicts the true label. A false positive (FP) is an incorrect prediction that is not the ‘None’ label. A False Negative was an incorrect prediction when the true label was not the ‘None’ label. If the prediction and true label mismatched and neither were the ‘None’ label, it was counted as both a FP and false negative. This overlap was properly dealt with when calculating metrics. For linking each possible link between tagged tokens was considered. TPs were predicted when they actually linked, FPs were predicted but not actually linked, and false negatives were not predicted but were actually linked.

Table 3 summarizes the models performance for each specific category using the mean of the *k*-fold validation. The overall average $F_{1}$ score across the predictors is 0.76. The individual $F_{1}$ scores for each predictor head were: Category: 0.80, Perturbing action: 0.76, Context: 0.74, Effect: 0.74, and Phenotype: 0.78. For the held-out test dataset, the average $F_{1}$ score for the predictors was 0.78, and for each head were: Category: 0.80, Perturbing action: 0.78, Context: 0.70, Effect: 0.80, and Phenotype: 0.82. Due to the small test set size, the consistent improvement of these scores over the validation ones is most likely due to the test set abstracts randomly being easier to tag.

**Table 3.** Tagging model performance per specific category using *k*-fold cross-validation average, with k=4.

Category	Precision	Recall	$F_{1}$ score
Perturbing action	0.743	0.786	0.764
Context	0.725	0.765	0.744
Effect	0.741	0.740	0.740
Phenotype	0.760	0.791	0.775

We also evaluated our linking model. For the *k*-fold validation the links between tags were predicted with a precision of 0.85, recall of 0.96, and $F_{1}$ score of 0.90. For the test set it achieved a precision of 0.95, recall of 0.94, and $F_{1}$ score of 0.94. For reference, using a co-occurrence baseline (i.e. where all entities that mentioned in the same sentence are assumed to be related) would result in an $F_{1}$ score of 0.42 due to the high number of FPs.

The results for the ablation study testing different pre-trained transformers baselines, and having each predictor with its own transformer, is given in Table 4. For brevity only the $F_{1}$ scores are given, using the average across all predictors for the tagging predictions.

**Table 4.** $F_{1}$ score for different architectures.^a^

Architecture	Tagging average		Linking	
	Validation	Testing	Validation	Testing
BioBERT	0.764	0.781	0.902	0.944
BioBERT Split	0.763	0.792	0.877	0.939
BioClinicalBERT	0.734	0.751	0.797	0.908
BERTBase	0.707	0.728	0.853	0.885

a Previously mentioned results are from the ‘BioBERT’-based model. ‘BioBERT Split’ denotes the variant where each tagging predictor has its own pre-trained transformer fine-tuned with it, and the link predictor is trained without the additional same-tag auxiliary classifier.

### 4.2 Extrinsic evaluation

To validate our methodology extrinsically, we carried out a case study using abstracts derived from a broad spectrum of 20 cell biology, genetics, cancer, and multidisciplinary journals. These were ‘Autophagy’, ‘Cancer Research’, ‘Genes and Development’, ‘Cell Death and Disease’, ‘Cell Death and Differentiation’, ‘Apoptosis’, ‘Cell’, ‘Cancer Cell’, ‘Nature’, ‘Nature Cell Biology’, ‘Nature Genetics’, ‘Nature Medicine’, ‘Nature Cancer’, ‘Science’, ‘Science Advances’, ‘eLife’, ‘Journal of Cell Biology’, ‘Journal of Cell Science’, ‘Cell Stem Cell’, and ‘Molecular Cell’. Using the PubMed Advanced Search tool, we selected abstracts that included any one of the selected journal titles as a journal name but excluded ‘review’ as a publication type. The first 10 000 abstracts listed were downloaded as a single text file (including metadata) and were used to test the performance of the model. Of these abstracts, 1526 contained one or more recognizable gene perturbations, with a mean of 1.91 (SD 1.21) gene perturbations per abstract.

In total, we extracted [we used BERN2 (Sung *et al.* 2022) to extract the mentions of genes] 2919 examples of genes that were each associated with a perturbing action. Many genes associated with perturbing actions were also linked to at least one phenotype and/or at least one context. Two hundred and thirty-one genes associated with a perturbing action were linked to a context, an effect and a phenotype, i.e. an example of every entity group, providing the complete set of information required to describe the function of a gene in a specific context.

#### 4.2.1 Precision of biological entity recognition and labelling

We selected the entities derived from the group of 231 case study genes that were associated with both a perturbing action and at least one context, one effect, and one phenotype. Some genes were associated with multiple entities in the Context, Effect, and/or Phenotype groups. To calculate the precision of entity recognition, correctly recognized entities were manually scored as TP and entities that should not have been extracted were scored as FP. Perturbing actions, Genes, Contexts, Effects, and Phenotypes were scored as separate groups. Values for precision for all groups were >0.9 (Table 5). Correctly recognized entities were then scored for entity labelling, where entities labelled with the correct assertion(s) were scored as TP and entities labelled with one or more incorrect assertions were labelled as FP. These data were used to calculate scores for the precision of entity labelling, generating values of >0.9 for every group (Table 5). These results show that our model is capable of a high degree of precision in both the recognition and correct labelling of relevant biomedical entities (Table 5). Of note, in the Contexts group, the model achieved high precision values for both entity recognition (0.99) and labelling (0.93), despite the diverse and often idiosyncratic vocabulary used to describe different body tissues, cell lines, and animal strains.

**Table 5.** Precision of entity recognition tagging and entity labelling.^a^

A: Precision of entity recognition tagging						
	PA	*G*	*E*	Ph	*C*	All
TP	214	231	230	214	229	1118
FP	17	0	1	17	2	37
Precision	**0.93**	**1.00**	**1.00**	**0.93**	**0.99**	**0.97**

B: Precision of entity labelling						
	PA	*G*	*E*	Ph	*C*	All
TP	205	214	223	202	213	1057
FP	9	17	7	12	16	61
Precision	**0.96**	**0.93**	**0.97**	**0.94**	**0.93**	**0.95**

a TP, true positive; FP, false positive; PA, perturbing actions; *G*, genes; *E*, effects; Ph, phenotypes; *C*, contexts. Precision (values in bold) refers to the number of true positives divided by the total number of positive predictions.

We used the same case study dataset to examine the distribution of labelled assertions within the different groups. The Perturbing actions group contained representatives from all six assertion groups (as described in Table 1). ‘Gene loss-of-function’ occurred with the highest frequency (91/231) and ‘Pharmacological augmentation’ occurred with the lowest frequency (6/231).

The Contexts and Phenotypes groups both contained representatives from most, but not all, assertion groups.

In the Contexts group, the most frequently occurring assertion was ‘Cells’ (50/231) and the second most frequently occurring was ‘Cells; Organism’ (32/231), i.e. studies which involved at least two experimental contexts. The assertion ‘Transformed cells’ (i.e. cancer cells) was highly represented both alone (19/231) and in combination with other assertions, such as ‘Cells’ (5/231). These results show that we were able to extract entities corresponding to a broad range of experimental contexts. The assertion ‘Patient’ was not represented, either alone or in combination with other assertions. This likely reflects the fact that human gene perturbation experiments are more often carried out in either cell lines or patient-derived cells, which would be represented within either the ‘Cells’ or ‘Transformed cells’ assertion groups.

Within the Phenotype group, the most frequently represented assertion terms were ‘Apoptosis’ (32/231), ‘Proliferation’ (23/231), and ‘Tumourigenesis’ (20/231). Some assertion terms, including ‘Anoikis’, ‘Ferroptosis’, and ‘Quiescence’ were not represented (though a proportion of these were represented within the larger, unfiltered case study dataset of entities associated with 2919 genes). These results suggest that our model could be further refined by training it on a larger and more diverse corpus of annotated abstracts containing a broader range of phenotype terms.

In cancer genomics, it is of particular interest to identify those genes, which can induce cell death in cancer cells but which do not induce death of normal, healthy cells. To explore the potential of our methodology to identify such genes, we focused on the cell death phenotype ‘Apoptosis’, which was represented by a high number of entities within our case study data. Within the filtered dataset, there were 52 perturbing action and gene mentions associated with ’Apoptosis’ (Table 6). These genes were connected to a broad range of context assertion terms, consistent with the fact that apoptosis is a common form of cell death that has been observed in a many different experimental models.

**Table 6.** Experimental gene perturbations associated with ‘Apoptosis’.

Total genes associated with phenotype ‘Apoptosis’	52
Genes associated with *in vitro* assertions (cells; transformed cells; *in vitro*; organoid)	45
Genes associated with *in vivo* assertions (organism; neoplasm, tissue/organ; *in vivo*; xenograft)	25
Overlap (genes associated with *in vitro* assertions that are also associated with *in vivo* assertions)	18
Genes associated with non-cancer assertions (organism, tissue/organ, cells, organoid, *in vitro*, *in vivo*)	41
Genes associated with cancer assertions (transformed cells; neoplasm; xenograft)	22
Overlap (genes associated with non-cancer assertions that are also associated with cancer assertions)	10

The case study sample size was too small to allow in-depth analysis of gene perturbations in individual contexts. To further analyse the results, we therefore grouped the apoptosis-associated genes in two separate ways; (i) genes associated with *in vitro* assertion terms compared with genes associated with *in vivo* assertion terms and (ii) genes associated with non-cancer assertion terms and compared genes associated with cancer assertion terms (as detailed in Table 1). Within each of these two pairs of groups, there were some genes that were present within only one group and others which were present within both groups (Table 6). For example, in the *in vitro* group versus *in vivo* group pairing, the genes BCL-6, FasL, and BAX were associated with apoptosis in *in vitro* contexts only, Tctp, PKD1, and Ldlr were associated with apoptosis in *in vivo* contexts only, and the genes Ang2, Foxo3a, mTOR, Cox-2, and Smad2 were associated with apoptosis in both *in vitro* and *in vivo* contexts. In the non-cancer group versus cancer group pairing, the genes Ang2, CD28, and Foxo3a were associated with apoptosis in non-cancer contexts only, Pim1, Hsp90, and EGFR were associated with apoptosis in cancer contexts only, and p53, RAB25, and E2F-1 were associated with apoptosis in both non-cancer and cancer contexts. Thus, our methodology was able to identify genes involved in the regulation of apoptosis in a range of contexts and, when applied to a larger sample size, could be a useful means to explore variation of regulatory mechanisms in different cell types and experimental contexts.

Although the small sample size used in our case study precludes drawing any specific biological inferences, our results provide a proof-of-principle demonstration of how our new methodology could be used to generate insights into an important real-world question in cancer genomics.

## 5 Discussion

Genomics research papers are superabundant within the biomedical domain of digital literature and their content is critically important both to the understanding of basic biological processes and to the design of new therapeutic strategies for cancer and other human diseases. There is an unmet need in biomedical TM for tailored methodologies that can systematically and accurately extract the most important details of genomics experiments from unstructured text, present them to researchers in an accessible form and thereby maximize the value of the existing knowledge base.

We have shown here that our new model can learn to recognize the five different classes of biological entity required to extract the results of genetics experiments from biomedical texts: genes, perturbing actions, effects, phenotypes, and contexts. We can automatically label the extracted entities with assertion terms that further define their meaning. Intrinsic evaluation has shown that our approach achieves a consistent performance of $F_{1}$ score between 0.75 and 0.79 across all categories. In addition, our methodology can accurately identify relations between the different groups of biological entities, with an $F_{1}$ score of 0.9. The accuracy of our work measures favourably against other comparable corpora, e.g. the Phenotype Gene Relations corpus (Sousa *et al.* 2019) and released models, has a maximum $F_{1}$ score of 0.68. Similarly, the model by Xing *et al.* (2018), which also extracts phenotype and genotype relationships from text has a best $F_{1}$ score of 0.67. In addition to the intrinsic evaluation, we have also demonstrated the accuracy of our approach in a real-world case study achieving a precision score over 0.9. Some key attributes of our model are that it is relatively lightweight and that it is able to run on a large quantity of data with a high level of accuracy.

The novel ability of our methodology to reliably and accurately recognize methods of gene perturbation and experimental contexts, which are both diverse and continuously evolving, can allow researchers to analyse the impact of genes in different experimental approaches and in different species and cell types. The methodology generalizes to various subdomains including basic cell biology, basic and applied cancer research, and multidisciplinary texts, enabling broad coverage of biomedical literature. It is also potentially adaptable to other related applications, e.g. the investigation of the impacts of genes or drugs on downstream gene expression or disease aetiologies.

The overall results of our evaluation demonstrate that our approach can accurately extract functional genomics relationships and relevant context from PubMed abstract text. In future work, we propose two key improvements. Firstly, it would be of high value to adapt our machine-learning models to work on full article texts, thereby providing access to a much greater volume of relevant information and context for extraction. Full-text classifier adaptation is possible and has been demonstrated in similar work (Oliveira Gonçalves *et al.* 2021, Gonçalves *et al.* 2022). Secondly, our methodology would benefit from linking the identified phenotypes to the recently available HPO (Groza *et al.* 2015). This will allow extracted phenotypes to be normalized to a standardized vocabulary, as well as enabling the integration of additional contextual information provided by HPO for disease phenotypes.

## 6 Conclusion

In this article, we have presented the first TM methodology that is specifically designed for extracting the contexts of genomics experiments. We have developed a NER model as well as a relation extraction model that can identify the key components of genomics experiments, including contexts, and have evaluated our models both intrinsically and extrinsically. Our approach leverages the latest developments in Natural Language Processing and Machine Learning and outperforms the $F_{1}$ scores of earlier models. We believe that our methodology has the potential to significantly enhance and accelerate researchers’ ability to access and contextualize genomics information compared to previously existing TM tools.

## Supplementary Material

btae021_Supplementary_Data
