# Supplementary material for: Text mining for contexts and relationships in cancer genomics literature
Source: Bioinformatics. 2024 Jan 22;40(1):btae021. doi: 10.1093/bioinformatics/btae021 (PMC10822582; doi:10.1093/bioinformatics/btae021)
Supplement: btae021_Supplementary_Data [file btae021_supplementary_data.pdf]

Supplementary Table 1. Characteristics of different forms of cell death.

Cell Death Type	Characteristics	Examples of known regulatory genes
**Apoptosis**	Regulated cell death that occurs in response to intrinsic or extrinsic stimuli, characterised by nuclear fragmentation and blebbing of the plasma membrane to form vesicles that are phagocytosed and degraded by lysosomes .	BCL2, BAX, BAK, CASP3, CASP9.
**Anoikis**	Subtype of apoptosis that is triggered by loss of contact.	BCL2, CASP9
**Autophagy**	'Self-eating' through the formation of double-membrane autophagosomes which are degraded by lysosomes.	ATG family genes, BECN1.
**Entosis**	Regulated non-apoptotic cell death by cell-in cell internalisation and lysosomal degradation.	TP53
**Ferroptosis**	Regulated cell death triggered by intracellular oxidative perturbations and inhibited by iron chelation.	GPX4
**Mitophagy**	Selective degradation or mitochondria by autophagy.	PINK1, PARKIN
**Necroptosis**	Form of regulated necrosis triggered by intracellular or extracellular homeostatic fluctuations.	RIPK1, RIPK3, MLKL
**Necrosis**	Cell death resulting from external trauma.	
**Oncosis**	Loss of membrane integrity that results in cell swelling followed by death without nuclear fragmentation.	TMEM123
**Pyroptosis**	Regulated cell death triggered by inflammasome activation.	CASP1
